# Nintedanib can be used safely and effectively for idiopathic pulmonary fibrosis with predicted forced vital capacity ≤ 50%: A multi-center retrospective analysis

**DOI:** 10.1371/journal.pone.0236935

**Published:** 2020-08-27

**Authors:** Satoru Senoo, Nobuaki Miyahara, Akihiko Taniguchi, Naohiro Oda, Junko Itano, Hisao Higo, Naofumi Hara, Hiromi Watanabe, Hirohisa Kano, Toshimitsu Suwaki, Yasuko Fuchimoto, Kazuhiro Kajimoto, Hirohisa Ichikawa, Kenichiro Kudo, Takuo Shibayama, Yasushi Tanimoto, Shoichi Kuyama, Arihiko Kanehiro, Yoshinobu Maeda, Katsuyuki Kiura, on behalf of Okayama Respiratory Disease Study Group (ORDSG)

**Affiliations:** 1 Department of Hematology, Oncology, and Respiratory Medicine, Okayama University Graduate School of Medicine, Dentistry and Pharmaceutical Sciences, Okayama, Japan; 2 Department of Medical Technology, Okayama University Graduate School of Health Sciences, Okayama, Japan; 3 Department of Allergy and Respiratory Medicine, Okayama University Hospital, Okayama, Japan; 4 Department of Respiratory Medicine, Okayama City Hospital, Okayama, Japan; 5 Department of Respiratory Medicine, Japan Organization of Occupational Health and Safety Okayama Rosai Hospital, Okayama, Japan; 6 Department of Respiratory Medicine, Japanese Red Cross Kobe Hospital, Kobe, Japan; 7 Department of Respiratory Medicine, KKR Takamatsu Hospital, Takamatsu, Japan; 8 Department of Respiratory Medicine, National Hospital Organization Okayama Medical Center, Okayama, Japan; 9 Department of Respiratory Medicine, National Hospital Organization Minami-Okayama Medical Center, Hayashima, Japan; 10 Department of Respiratory Medicine, National Hospital Organization Iwakuni Clinical Center, Iwakuni, Japan; California Health Sciences University, UNITED STATES

## Abstract

**Background:**

Nintedanib is a multi-kinase inhibitor approved for idiopathic pulmonary fibrosis (IPF); however, its efficacy and safety for patients with IPF and restricted pulmonary function remain unclear. Therefore, the objective of this study was to determine the efficacy and safety of nintedanib for patients with IPF and forced vital capacity (FVC) ≤ 50%.

**Methods:**

This was a multi-center retrospective study performed by the Okayama Respiratory Disease Study Group. Patients were allocated into FVC ≤ 50% and FVC > 50% groups based on their predicted FVC. The primary endpoints were FVC changes from baseline after 6 and 12 months.

**Results:**

45 patients were eligible for the study. 18 patients had FVC ≤ 50%, and 27 patients had FVC > 50%. Overall, 31 and 19 patients underwent pulmonary function tests at 6 and 12 months after initiating nintedanib, respectively. FVC changes from baseline at 6 and 12 months after initiating nintedanib were comparable between the two groups. Adverse events were seen in all patients, and the rates of patients who discontinued nintedanib were also comparable (38.9% vs. 37.0%, p = 1.000). Multiple regression analysis showed that age and forced expiratory volume in 1 second (FEV1)/FVC were negatively correlated with changes in FVC at 6 months after initiating nintedanib.

**Conclusions:**

Our data suggest that nintedanib can be a useful agent for IPF patients, including those with a low FVC, and that age and FEV1/FVC are predictive markers for changes in FVC following nintedanib treatment.

## Introduction

Idiopathic pulmonary fibrosis (IPF) is characterized by chronic and progressive fibrosis of the lung of unknown etiology [1]. The prognosis of patients with IPF is generally poor, with a median survival time of 2–3 years [2]. Nintedanib is a multi-kinase inhibitor targeting receptors of vascular epithelial growth factors, platelet-derived growth factors, and fibroblast growth factors. In Japan, it was approved in 2015 for IPF, based on the results of the INPULSIS-1 and -2 trials. The INPULSIS trials were multicenter double-blind randomized studies. The primary endpoint of these studies was the annual decline in forced vital capacity (FVC), which was better in the nintedanib group than that in the placebo group [3]. Nintedanib has now been recommended for treatment according to the IPF guidelines [4–5].

Patients in the INPULSIS trials who had predicted FVC ≤ 50% were excluded, such that it is unclear whether nintedanib is safe and effective in patients with limited lung function. In the INPULSIS-ON trial, which is an open-label extension of the INPULSIS trials, patients with baseline FVC ≤ 50% were the participants; the safety and efficacy of nintedanib for those patients was demonstrated in an interim analysis [6]. However, only patients whose FVC values were originally > 50% of the predicted values were eligible for the INPULSIS trials; thus, the real-world benefit of nintedanib remains unclear for patients with predicted FVC ≤ 50%. Although some studies exploring this have been reported [7–9], the evidence is insufficient.

Therefore, the aim of this study was to investigate the safety and the efficacy of nintedanib for patients with IPF and a predicted FVC ≤ 50%.

Our study suggests that nintedanib can be safely administered to patients with IPF and FVC ≤ 50%. In addition, the change in FVC was comparable between the FVC ≤ 50% and FVC > 50% groups.

## Patients and methods

### Patients

All enrolled subjects were IPF patients taking nintedanib and treated at Okayama University Hospital, Okayama City Hospital, Japan Organization of Occupational Health and Safety Okayama Rosai Hospital, Japanese Red Cross Kobe Hospital, KKR Takamatsu Hospital, National Hospital Organization Okayama Medical Center, National Hospital Organization Minami-Okayama Medical Center, or the National Hospital Organization Iwakuni Clinical Center. IPF was clinically diagnosed based on the 2011 ATS/JRS/ERS/ALAT guidelines [10]. Patients who met the eligibility criteria of the INPULSIS trials, based on radiological findings, were included, i.e., presence of a reticular abnormality and traction bronchiectasis consistent with fibrosis showing basal and peripheral predominance; absence of atypical features, specifically nodules and consolidation, and ground glass opacity (where present) that was less extensive than reticular opacity [3]. All patients underwent high-resolution computed tomography at the time of diagnosis, and as well as at least one pulmonary function test between 3 months before and 1 week after initiating nintedanib. The patients began to use nintedanib between August 2015 and September 2017.

### Study design

Clinical data were collected retrospectively from the patients’ medical records. The primary endpoint was a decline in FVC at 6 and 12 months after initiating nintedanib. This study adhered to the principles of the Declaration of Helsinki and was approved by the Institutional Review Board (IRB) of Okayama University Hospital (no. 1710–038; approved on October 13, 2017), and all of the participating hospitals. All clinical data collected from medical records were anonymized, and their confidentiality was ensured. The IRB waived the requirement for written informed consent because this retrospective study provided information disclosure to the patients with a chance to refuse to participate in the study (opt-out method).

### Statistical analysis

Continuous variables are presented as median and range, and categorical variables as numbers with percentages. Overall survival (OS) was estimated by the Kaplan–Meier method. The significance of between group differences was assessed by the Mann–Whitney test for continuous variables and Fisher’s exact test for categorical variables. The paired t-test was used to compare paired samples. Before performing the multiple regression analysis, the Kolmogorov–Smirnov test was used to check whether the distribution of the FVC change data was normal. Pearson’s correlation coefficients were calculated for each explanatory variable. A multiple regression analysis was performed with explanatory variables of age and sex, and the two variables with the two lowest p-values (except age). A p-value < 0.05 was considered significant. All statistical analyses were performed with EZR (Saitama Medical Center, Jichi Medical University, Saitama, Japan) [11], which is a graphical user interface for R version 3.4.1 (R Foundation for Statistical Computing, Vienna, Austria).

## Results

### Patient characteristics

The patient characteristics are summarized in Table 1. 45 patients were enrolled in this study (89% male) with age ranging from 41 to 86 years (median, 69 years). Of the patients, 1 was a current smoker, 37 were former smokers, and 7 patients had never smoked.

**Table 1:** Patient characteristics.

	Total	FVC ≤ 50% predicted	FVC > 50% predicted	P-value
	n = 45	n = 18	n = 27	
Age, years, median (range)	69 (41–86)	68 (41–80)	70 (53–86)	0.296
Sex, male/female, n (%)	40 (88.9%)/5 (11.1%)	15 (83.3%)/3 (16.7%)	25 (92.6%)/2 (7.4%)	0.375
Smoking history				
Current smoker, n (%)	1 (2.2%)	0 (0.0%)	1 (3.7%)	0.651
Former smoker, n (%)	37 (82.2%)	14 (77.8%)	23 (85.2%)	
Never smoker, n (%)	7 (15.6%)	4 (22.2%)	3 (11.1%)	
Pack-years, median (range)	30.0 (0–126)	12.5 (0–70.5)	38.0 (0–126)	**0.018**
Body mass index, median (range)	22.6 (13.0–30.5)	21.9 (13.0–30.5)	23.1 (18.1–24.2)	0.412
Body surface area, m^2^, median (range)	1.65 (1.15–1.96)	1.65 (1.15–1.95)	1.65 (1.29–1.96)	0.804
FVC, ml, median (range)	1950 (510–3660)	1270 (510–1950)	2580 (1180–3660)	**<0.001**
FVC, % predicted, median (range)	60.5 (23.5–92.4)	40.5 (23.5–49.4)	69.4 (51.3–92.4)	**-**
FEV1/FVC, %, median (range)	87.6 (69.8–125.5)	93.3 (81.5–100)	85.3 (69.8–96.6)	**<0.001**
performed D_LCO_, n (%)	25 (55.6%)	8 (44.4%)	17 (63.0%)	0.241
D_LCO_, %, median*	38.3 (5.2–77.1)	31.2 (5.2–57.1)	47.6 (16.5–77.1)	**0.031**
GAP score, median*	2–6 (5)	4–6 (5)	2–6 (4)	0.053
Performed 6-min walk test, n (%)	24 (53.3%)	11 (61.1%)	13 (48.1%)	0.757
6-min walk distance, m, median (range)**	317.5 (30–550)	300 (30–400)	360 (60–550)	0.234
Disease severity staging for IPF in Japan [12,13], median (range) **	4 (1–4)	4 (1–4)	4 (1–4)	0.642
Time since IPF diagnosis	407 (8–2691)	392 (8–2673)	438 (21–2691)	0.847
days, median (range)				
Long-term oxygen therapy, n (%)	24 (53.3%)	12 (66.7%)	12 (44.4%)	0.223
Radiological pattern, n (%)				
UIP pattern	31 (68.9%)	9 (50.0%)	22 (81.5%)	**0.038**
possible UIP pattern	6 (13.3%)	5 (27.8%)	1 (3.7%)	
inconsistent with UIP pattern	8 (17.8%)	4 (22.2%)	4 (14.8%)	
Presence of emphysema, n (%)	4 (8.9%)	3 (16.7%)	1 (3.7%)	0.286
PA/Ao > 0.9, n (%)	21 (46.7%)	9 (50.0%)	12 (44.4%)	0.763
Performed echocardiography, n (%)	14 (31.1%)	5 (29.4%)	9 (33.3%)	0.753
TRPG, mmHg, median (range)	35 (16–61)	30 (20–49)	41 (16–61)	0.461
performed SLB, n (%)	11 (24.4%)	5 (29.4%)	6 (23.1%)	0.732
UIP pattern, n (% of performed SLB)	11 (100%)	5 (100%)	6 (100%)	1.000
Previous pirfenidone use, n (%)	16 (35.6%)	6 (33.3%)	10 (37.0%)	1.000
KL-6, median (range)	1204 (238–3520)	1160.5 (334–3520)	1204 (238–2716)	0.772

Continuous variables are presented as median and range, and categorical variables are presented as numbers with percentages. The significance of the between-group differences was assessed using the Mann–Whitney test for continuous variables and Fisher’s exact test for categorical variables. Abbreviations: FVC, forced vital capacity; FEV1, forced expiratory volume in 1 second; D_LCO_, diffusing capacity for carbon monoxide; IPF, idiopathic pulmonary fibrosis; UIP, usual interstitial pneumonia; PA/Ao, ratio of pulmonary arterial and aortic diameters; TRPG, tricuspid regurgitation pressure gradient; TRPG, tricuspid regurgitation pressure gradient; SLB, surgical lung biopsy.

*Data from patients who underwent D_LCO_ testing.

**Data from patients who underwent the 6-min walk test

**Fig 1:**
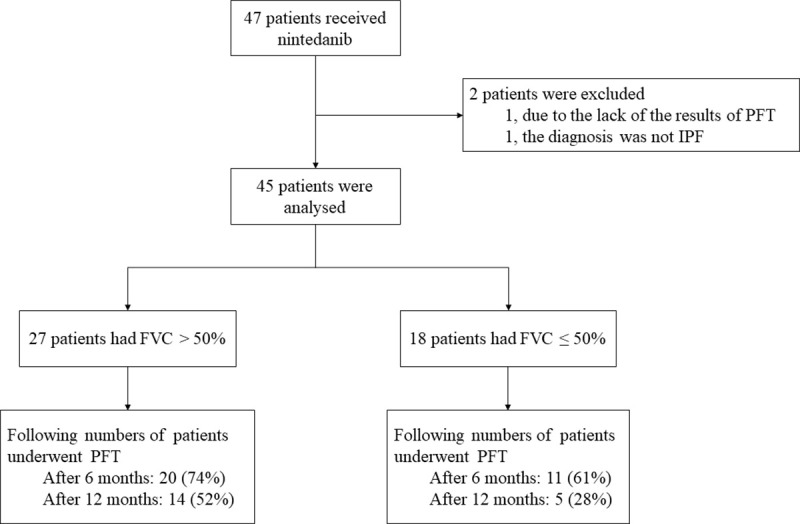
Study flow chart.

Of the 45 patients, 18 had a predicted FVC ≤ 50%, and 27 had an FVC > 50% (Fig 1). The number of pack-years was significantly higher, and the forced expiratory volume in 1 second (FEV1)/FVC was significantly lower, in the FVC > 50% group. Computed tomography revealed that most of the patients in the FVC > 50% group had a typical interstitial pneumonia (UIP) pattern (81.9%), compared to only 50% of those in the FVC ≤ 50% group. Emphysema tended to be more frequently observed in the FVC ≤ 50% group (3 [16.7%]) than the FVC > 50% (1 [3.7%]) group, but the difference was not statistically significant.

The baseline D_LCO_ was significantly lower in the FVC ≤ 50% group than in the FVC > 50% group. Patients with pulmonary hypertension exhibit low D_LCO_ values; we thus explored the pulmonary hypertension statuses of both groups. As shown in Table 1, in patients who underwent echocardiography, the tricuspid regurgitation pressure gradient (TRPG) (used to estimate pulmonary artery pressure) was comparable between the two groups. We also assessed the pulmonary arterial:aortic diameter ratio; a value > 0.9 is suggestive of pulmonary hypertension. The proportions of patients with pulmonary arterial: aortic diameter ratio > 0.9 were similar in the two groups. Thus, the difference in the D_LCO_ values between the two groups was not attributable to pulmonary hypertension in the FVC ≤ 50% group.

No significant group differences were observed in age, sex, smoking history, body mass index (BMI), body surface area, time since diagnosis of IPF, or the rate of patients who received home oxygen therapy.

### Clinical course

The clinical course data are shown in Table 2. The median observation period was 441 days in the overall population. The median OS time since initiating nintedanib was 736 days. Adverse events were observed in all patients. The first- and second-most frequent adverse events were an increase of liver enzymes and diarrhea, respectively. Of the patients, 31 and 19 had available data for pulmonary function tests performed 6 months and 12 months after the initiation of nintedanib, respectively. The median observation period in the FVC ≤ 50% group was 281.5 days.

**Table 2:** Outcomes and adverse events.

	Total	FVC ≤ 50% predicted	FVC > 50% predicted	P-value
	n = 45	n = 18	n = 27	
**Outcomes**				
Medication period in days, median (range)	387 (18–1056)	195.5 (18–742)	414 (36–1,056)	0.179
Observation period in days, median (range)	441 (19–1056)	281.5 (19–742)	449 (74–1,056)	0.056
Overall survival since initiation of nintedanib in days, median (95% CI)	736 (515–779)	650 (135–NA)	742 (492–NA)	**0.042**
**Adverse events**				
Any, n (%)	45 (100%)	18 (100%)	27 (100%)	1.000
Diarrhea, n (%)	22 (48.8%)	6 (33.3%)	16 (59.3%)	0.130
Nausea, n (%)	7 (15.5%)	3 (16.7%)	4 (14.8%)	1.000
Nasopharyngitis, n (%)	4 (8.9%)	1 (5.6%)	3 (11.1%)	0.640
Bronchitis, n (%)	8 (17.8%)	4 (22.2%)	4 (14.8%)	0.694
Weight loss, n (%)	4 (8.9%)	2 (11.1%)	2 (7.4%)	1.000
Fatigue, n (%)	13 (28.9%)	5 (27.8%)	8 (29.6%)	1.000
Decreased appetite, n (%)	13 (28.9%)	7 (38.9%)	6 (22.2%)	0.317
Liver enzyme elevation, n (%)	28 (62.2%)	11 (61.1%)	17 (63.0%)	1.000
Dose-down or discontinuation due to adverse events, n (%)	23 (51.1%)	9 (50.0%)	14 (51.9%)	1.000
Discontinuation due to adverse events, n (%)	17 (37.8%)	7 (38.9%)	10 (37.0%)	1.000

Continuous variables are presented as median and range, and categorical variables are presented as numbers with percentages. The log-rank test was used to determine significant differences between groups, except for medication and observation periods (Mann–Whitney test), and adverse events (Fisher’s exact test).

**Fig 2:**
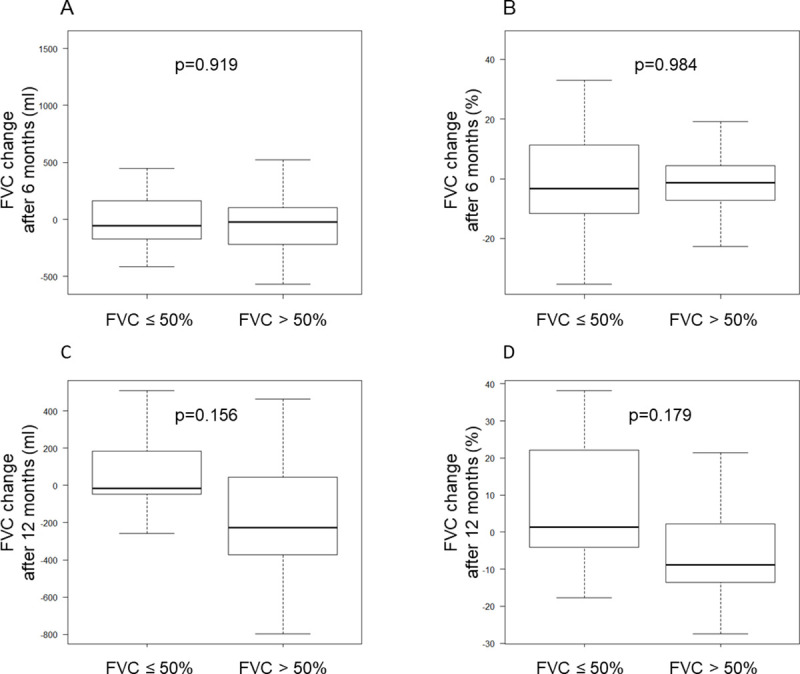
Differences in the change in Forced Vital Capacity (FVC) between patients with FVC > 50% vs. ≤50%. A and C show the decline in FVC from baseline over 6 and 12 months, respectively. B and D show the rate of FVC decline from baseline over 6 and 12 months, respectively.

**Fig 3:**
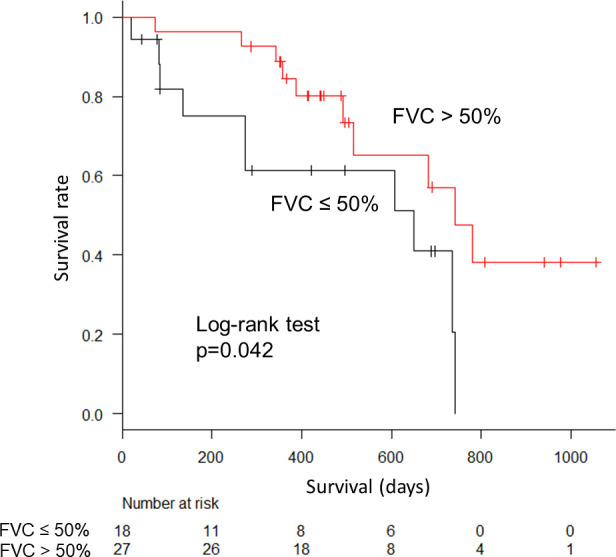
Kaplan–Meier curve from initiation of nintedanib. The forced vital capacity (FVC) > 50% group survived longer than the FVC ≤ 50% group.

FVC changes from baseline to 6 months after initiating nintedanib did not differ significantly between the FVC > 50% and ≤ 50% groups (Fig 2A and 2B), and data were available in 74% and 61% of the patients, respectively. The FVC change after 12 months showed a similar tendency (Fig 2C and 2D); however, fewer data were available in the FVC ≤ 50% group than in the FVC > 50% group (28% and 52%, respectively). OS was significantly better in the FVC > 50% group compared to the FVC ≤ 50% group (p = 0.042; Fig 3). The incidence of adverse events was comparable between the groups, where almost half of the patients were required to reduce the dose or discontinue the agent (Table 2).

Patients who underwent echocardiography were divided into TRPG ≥ 37 and TRPG < 37 mmHg groups; survival was compared between groups. Patients with higher TRPG values exhibited somewhat poorer survival than did patients with lower TRPG values, but this difference was not statistically significant (S1 Fig).

**Fig 4:**
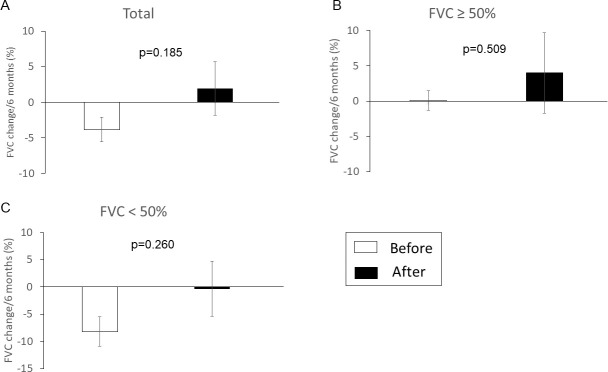
FVC changes after initiation of nintedanib. We compared the changes in predicted FVC values after initiation of nintedanib; the values tended to increase in both the FVC > 50% and FVC ≤ 50% groups. Values are expressed as the means ± standard error of the means. Before: before initiation of nintedanib. After: after initiation of nintedanib.

We explored whether nintedanib reduced the declines in FVC (Fig 4). We assessed the relative changes in predicted FVC values at 6 months before initiation of nintedanib, as well as immediately prior to drug initiation, in the FVC > 50% and FVC ≤ 50% groups. 10 patients in the FVC > 50% group and 9 patients in the FVC ≤ 50% group underwent pulmonary function testing prior to initiation of nintedanib; we compared these data to the FVCs after initiation. Fig 4 shows that the changes in predicted FVC values after initiation of nintedanib tended to increase compared to those before initiation of nintedanib in both the FVC > 50% and FVC ≤ 50% groups.

**Fig 5:**
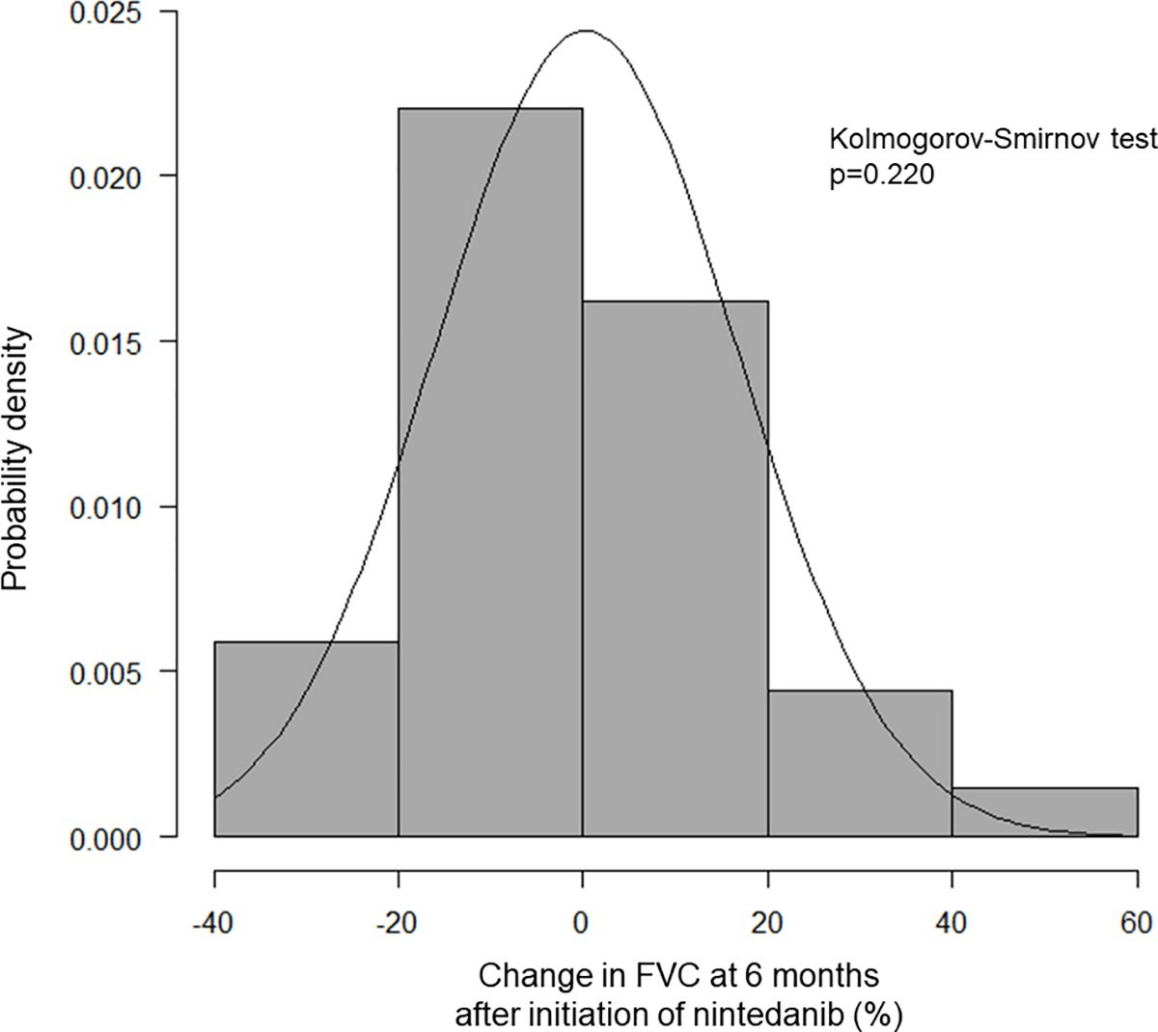
Kolmogorov–Smirnov test to determine the normality of the data for the relative change in predicted FVC from baseline. The data were normally distributed.

In this study, we determined predictors of a change in FVC. Before the analysis, the normality of the relative change in predicted FVC data was tested by the Kolmogorov–Smirnov test, which revealed a normal distribution (Fig 5). Pearson’s correlation coefficients were calculated for each explanatory variable. The univariate analysis showed that age and IPF disease severity [12,13] were significantly correlated with the relative change in predicted FVC, but BMI, body surface area, FEV1/FVC, pack-years, FVC, predicted FVC, predicted diffusing capacity for carbon monoxide (D_LCO_), time since diagnosis of IPF, and KL-6 level were not.

A multiple regression analysis was performed, including age, sex, and the two variables with the two lowest p-values (except age) as explanatory variables (FEV1/FVC and BMI). Age and FEV1/FVC were negatively correlated with the relative change in predicted FVC 6 months after initiating nintedanib (Table 3).

**Table 3:** Pearson’s correlation of variables with predicted FVC change at 6 months and results of multiple regression analysis.

Variables	Correlation coefficient	P-value
Age, years	−0.551	**0.001**
Sex (male: 1, female: 0)	−0.020	0.914
Pack-years	0.156	0.402
Body mass index	0.254	0.167
Body surface area, m^2^	0.245	0.184
FVC, ml	0.189	0.309
FVC, % predicted	0.054	0.772
FEV1/FVC, %	−0.274	0.136
Time since IPF diagnosis, days	−0.192	0.302
Long-term oxygen therapy (yes: 1, no: 0)	0.234	0.205
Previous pirfenidone use (yes: 1, no: 0)	0.055	0.770
**Multivariate analysis**	**B**	**P-value**
Age, years	−1.238	<0.001
Sex (male: 1, female: 0)	−13.29	0.068
Body mass index	1.234	0.125
FEV1/FVC, %	−0.888	<0.001

Pearson’s correlation coefficient was calculated for each explanatory variable, and multiple regression analyses included age, sex, body mass index, and FEV1/FVC; the two variables with the two lowest p-values (except age) were used as explanatory variables. Abbreviations: FVC, forced vital capacity; FEV1, forced expiratory volume in 1 second; IPF, idiopathic pulmonary fibrosis.

## Discussion

In this study, we compared the decline in FVC and incidence rates of adverse events between Japanese patients with IPF and FVC > 50% versus FVC ≤ 50%. The changes in predicted FVC and incidence of adverse events did not differ significantly between the two groups at 6 or 12 months after initiating nintedanib.

One of the eligibility criteria for the INPULSIS trials [3] was predicted FVC > 50%, and patients with limited pulmonary function were excluded. An interim analysis of the INPULSIS-ON trial [6] revealed the efficacy and the safety of nintedanib in patients with a FVC ≤ 50% for the first time; however, patients who participated in the INPULSIS-ON trial originally had a FVC > 50% when registered for the INPULSIS trials. In other words, the data from INPULSIS-ON trial might not have reflected real-world data. The results from our study revealed that nintedanib has efficacy for patients with a low FVC, and also provided protection against adverse events.

In our study, the change in FVC did not differ significantly between patients with FVC ≤ 50% and those with FVC > 50% (−56 mL/6 months vs. −22 mL/6 months, respectively; p = 0.919). The patient characteristics were similar between the groups, except that the FVC > 50% group had higher pack-years and lower FEV1/FVC values than those in the FVC ≤ 50% group. Nintedanib tended to reduce the decline in FVC, even in the FVC ≤ 50% group. Patients who received nintedanib exhibited smaller FVC reductions, compared to patients not prescribed the drug; this was consistent with the findings of previous studies [6,8,9]. The effects of nintedanib in the FVC ≤ 50% group were also comparable to those of previous studies [6,8,9]. In the interim analysis of the INPULSIS-ON trial, nintedanib was efficacious even in the FVC ≤ 50% group, as seen in the FVC > 50% group [6]. Our data and a previous report indicate that nintedanib is effective even in patients with a lower FVC.

No group differences in adverse events or the rate of patients requiring a dose reduction or discontinuation of nintedanib were observed. In the interim analysis of the INPULSIS-ON trial, patients who experienced adverse events leading to treatment discontinuation were more frequent in the FVC ≤ 50% group [6]. The same tendency has been reported in other studies [8,9]. We consider that nintedanib must be used carefully, but can be used safely even in patients with a low FVC.

Here, we discuss the reason why the FVC > 50% group had higher pack-years and lower FEV1/FVC values than those in the FVC ≤ 50% group in our study. Although most of the patients had the UIP radiological pattern, they may have had a complex pathophysiology comprising chronic obstructive pulmonary disease (COPD) caused by smoking; patients with IPF and COPD may have a FEV1/FVC ratio in the normal range, an entity known as combined pulmonary fibrosis and emphysema (CPFE) [14]. Given that only 1 patient in the FVC > 50% group had emphysema, some of the patients may have had chronic bronchitis, which is one of the phenotypes of COPD in which emphysema does not develop.

In this study, we determined predictors of a change in FVC in an exploratory analysis. In a previous report, predictors of a change in FVC were unclear in nintedanib responders and non-responders [15]. We performed a multiple regression analysis on the relative change in predicted FVC as the outcome variable; age and FEV1/FVC were negatively correlated with the change in FVC at 6 months after initiating nintedanib. In other words, the patients with higher age and higher FEV1/FVC experienced a more severe decline in FVC. Several studies have demonstrated that a lower FEV1/FVC is associated with a better prognosis in patients with IPF [16–18]; however, the reason remains unclear. One suggested mechanism is that a lower FEV1/FVC might reflect a complex pathophysiology including COPD as mentioned above. A lower FEV1/FVC suggests the existence of CPFE, making an FVC apparently higher; however, only 1 of our patients was diagnosed with COPD, and only 3 (16.7%) and 1 (3.7%) patients with FVC ≤ 50% and > 50%, respectively, exhibited emphysematous changes in radiological assessments (Table 1). In a previous study that reported that lower FEV1/FVC indicates a better IPF prognosis, only 8.2% of lower FEV1/FVC cases had emphysema [18]. Therefore, that study and the present study suggest that FEV1/FVC is an important prognostic factor regardless of the presence or absence of emphysema. A clinical trial of predictors of nintedanib efficacy is currently ongoing (NCT 02788474).

Some limitations of this study should be discussed. First, it used a retrospective design and the sample size was small. Second, some patients did not undergo D_LCO_ testing. Patients with D_LCO_ ≤ 30% should be regarded as having low pulmonary function, as they were excluded from INPULSIS trials. However, we did not allocate them to the low pulmonary function group, as > 40% of all patients had an unknown D_LCO_. Third, not all of the patients were able to undergo pulmonary function tests at 6 and 12 months after initiating nintedanib, and the follow-up period was shorter than 12 months in the FVC ≤ 50% group (281.5 days) because of their poorer general condition and worse OS, as shown in Fig 3; therefore, the potential bias in our study should be given due consideration.

## Conclusions

Nintedanib is a good choice for treating IPF, even in patients with FVC ≤ 50%, and that the age and FEV1/FVC are predictive markers of the efficacy of nintedanib. However, the evidence may at present be insufficient; thus, additional studies are warranted.

## Supporting information

S1 Fig Kaplan–Meier curves for survival after initiation of nintedanib. 7 patients with higher TRPG values tended to exhibit poorer survival than patients with lower TRPG values (also 7 patients), but this difference was not statistically significant.
(TIF)

S1 Appendix (XLSX)

S1 Data (XLSX)
