# Supplementary figures and images for: Nintedanib can be used safely and effectively for idiopathic pulmonary fibrosis with predicted forced vital capacity ≤ 50%: A multi-center retrospective analysis
Source: PLoS One. 2020 Aug 27;15(8):e0236935. doi: 10.1371/journal.pone.0236935 (PMC7451511; doi:10.1371/journal.pone.0236935)

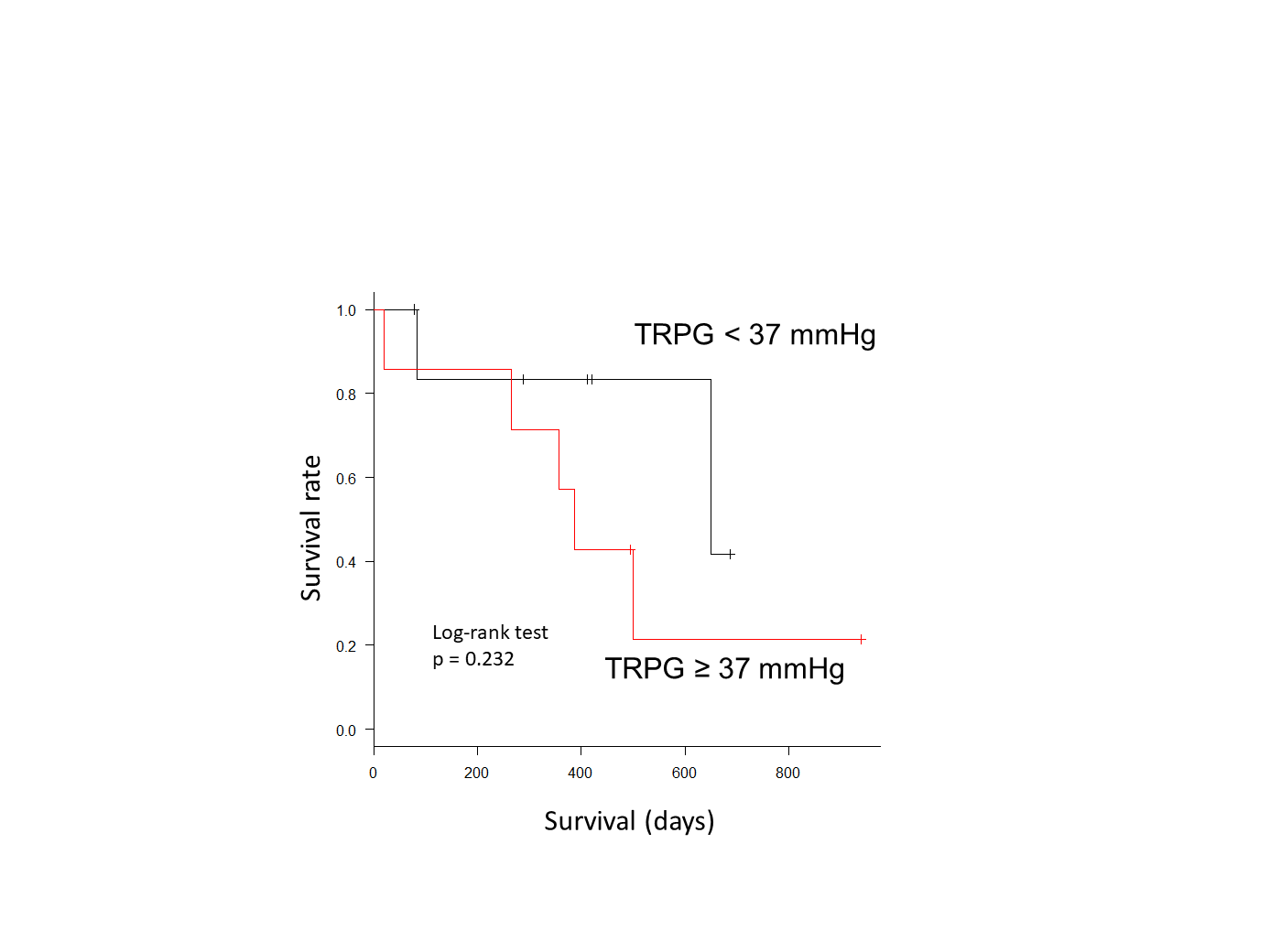

Supplement: S1 Fig — 7 patients with higher TRPG values tended to exhibit poorer survival than patients with lower TRPG values (also 7 patients), but this difference was not statistically significant. (TIF) [file pone.0236935.s001.tif]
